# Letter to the Editor: ‘Rare but relevant: Genetic liver disease in the general medical setting’

**DOI:** 10.1016/j.clinme.2026.100565

**Published:** 2026-03-07

**Authors:** Eamon P. McCarron

**Affiliations:** a Adult Inherited Metabolic Disorders, Northern General Hospital, Sheffield Teaching Hospitals NHS Foundation Trust, England, UK; b Faculty of Health, University of Sheffield, Sheffield, UK

Dear Editor,

I read with interest the article by Allouni and Ala^1^ on genetic liver disease in general medical settings. I strongly support their conclusion that, although individually rare, these conditions are clinically significant and frequently under-recognised due to their non-specific presentations. Early identification can redirect clinical management, facilitate targeted treatment and enable cascade testing, particularly as genomic services expand across the NHS.

From the perspective of inherited metabolic disorders (IMDs), the diagnostic framework outlined in the article is highly relevant. While Wilson disease, haemochromatosis and alpha-1 antitrypsin deficiency are well-established examples, a broader spectrum of IMDs is increasingly recognised in adult hepatology. These include lysosomal acid lipase deficiency (LAL-D), familial hypobetalipoproteinaemia, urea cycle disorders, mitochondrial hepatopathies and hepatic glycogen storage disorders, many of which are now considered treatable. Presentations are often subtle, episodic or multisystemic, and may be mislabelled as cryptogenic liver disease or misattributed to lifestyle factors. Awareness among generalists and hepatologists remains limited, despite growing evidence that early recognition and diagnosis of IMDs in general can significantly alter clinical trajectory.^2^

Recent studies show that genomic testing in adults with unexplained liver disease yields a molecular diagnosis in up to 25% of cases, frequently identifying IMDs.^3^^,^^4^ Notably, enzyme replacement therapy with sebelipase alfa is now available for LAL-D, a treatable and under-recognised cause of cryptogenic fatty liver disease. LAL-D may underlie up to 2–3% of adults with cryptogenic steatosis or cirrhosis and responds to therapy with sustained biochemical and histological improvement.^5^ Structured diagnostic pathways combining targeted biochemical testing and next-generation sequencing are increasingly accessible through the NHS Genomic Medicine Service and offer substantial clinical benefit.

The accompanying Table 1 highlights examples of IMDs categorised by pattern of liver disease. Improving recognition of these conditions, and embedding genomic and metabolic investigations into clinical practice, will help reduce diagnostic delays, enable targeted treatment, and support accurate diagnosis and prognostication in adults with unexplained liver disease.

**Table 1:** Examples of inherited metabolic disorders causing liver disease: pattern-specific disorders and clinical features.

Category	Disorders	Clinical features
Structural liver disease	Hepatic glycogen storage disorders (eg GSD I, III)	Hepatomegaly, hypoglycaemia, cirrhosis, adenomas, hepatocellular carcinoma, elevated transaminases, hyperuricaemia, hypertriglyceridaemia
	Lysosomal storage disorders (eg Gaucher, Niemann–Pick B) Lysosomal acid lipase deficiency (LAL-D)	Hepatosplenomegaly, cytopenias, lipid-laden macrophages, bone marrow involvement Hepatosplenomegaly, microvesicular steatosis, dyslipidaemia (low HDL, high TG), early cirrhosis
No structural liver disease	Organic acidaemias (eg propionic, methylmalonic acidaemia)	Metabolic acidosis, hyperammonaemia, elevated transaminases, neurologic signs
	Maple syrup urine disease	Ketoacidosis, hyperleucinaemia, elevated transaminases
	Urea cycle disorders (eg ornithine transcarbamylase deficiency (OTC) deficiency)	Hyperammonaemia, protein aversion, encephalopathy, elevated transaminases
	Tyrosinaemia type 1	Neurological crises, renal tubular dysfunction, elevated transaminases, raised alpha-fetoprotein (AFP), chronic disease can lead to cirrhosis, hepatocellular carcinoma
Fatty liver disease	Familial hypobetalipoproteinaemia, abetalipoproteinaemia	Low LDL-C, fat-soluble vitamin deficiency, steatosis, acanthocytosis
	Citrin deficiency	Intermittent transaminitis, postprandial symptoms, hyperammonaemia, steatosis
Cholestatic liver disease	Bile acid synthesis defects (eg HSD3B7, AKR1D1)	Neonatal cholestasis, low/normal GGT, fat-soluble vitamin deficiency, failure to thrive
	Erythropoietic protoporphyria (EPP)	Photosensitivity, cholestasis, elevated protoporphyrin, risk of hepatic fibrosis
Liver + multisystem involvement	Alagille syndrome	Cholestasis, bile duct paucity, cirrhosis, cardiac/facial anomalies, butterfly vertebrae
	Mitochondrial hepatopathies (eg POLG, DGUOK, MPV17 mutations)	Lactic acidosis, neurologic regression, hypoglycaemia, acute liver failure (±muscle/brain involvement)

## CRediT authorship contribution statement

**Eamon P. McCarron:** Conceptualization, Writing – original draft, Writing – review & editing.

## Declaration of competing interest

None.
